# Human in vitro neurocardiac coculture (*iv* NCC) assay development for evaluating cardiac contractility modulation

**DOI:** 10.14814/phy2.15498

**Published:** 2022-11-02

**Authors:** Akshay Narkar, Tromondae K. Feaster, Maura Casciola, Ksenia Blinova

**Affiliations:** ^1^ Center for Devices and Radiological Health US Food and Drug Administration Silver Spring Maryland USA

**Keywords:** cardiac contractility modulation CCM, cardiomyocytes, human induced pluripotent stem cells, neurocardiac coculture, neurons

## Abstract

Two of the most prominent organ systems, the nervous and the cardiovascular systems, are intricately connected to maintain homeostasis in mammals. Recent years have shown tremendous efforts toward therapeutic modulation of cardiac contractility and electrophysiology by electrical stimulation. Neuronal innervation and cardiac ganglia regulation are often overlooked when developing in vitro models for cardiac devices, but it is likely that peripheral nervous system plays a role in the clinical effects. We developed an in vitro neurocardiac coculture (*iv*NCC) model system to study cardiac and neuronal interplay using human induced pluripotent stem cell (hiPSC) technology. We demonstrated significant expression and colocalization of cardiac markers including troponin, α‐actinin, and neuronal marker peripherin in neurocardiac coculture. To assess functional coupling between the cardiomyocytes and neurons, we evaluated nicotine‐induced β‐adrenergic norepinephrine effect and found beat rate was significantly increased in *iv*NCC as compared to monoculture alone. The developed platform was used as a nonclinical model for the assessment of cardiac medical devices that deliver nonexcitatory electrical pulses to the heart during the absolute refractory period of the cardiac cycle, that is, cardiac contractility modulation (CCM) therapy. Robust coculture response was observed at 14 V/cm (5 V, 64 mA), monophasic, 2 ms pulse duration for pacing and 20 V/cm (7 V, 90 mA) phase amplitude, biphasic, 5.14 ms pulse duration for CCM. We observed that the CCM effect and kinetics were more pronounced in coculture as compared to cardiac monoculture, supporting a hypothesis that some part of CCM mechanism of action can be attributed to peripheral nervous system stimulation. This study provides novel characterization of CCM effects on hiPSC‐derived neurocardiac cocultures. This innervated human heart model can be further extended to investigate arrhythmic mechanisms, neurocardiac safety, and toxicity post‐chronic exposure to materials, drugs, and medical devices. We present data on acute CCM electrical stimulation effects on a functional and optimized coculture using commercially available hiPSC‐derived cardiomyocytes and neurons. Moreover, this study provides an in vitro human heart model to evaluate neuronal innervation and cardiac ganglia regulation of contractility by applying CCM pulse parameters that closely resemble clinical setting. This *iv*NCC platform provides a potential tool for investigating aspects of cardiac and neurological device safety and performance.

## Graphical abstract

Human innervated heart model combining induced pluripotent stem cell‐derived cardiomyocytes and neurons was developed to assess cardiac contractility modulation (CCM) stimulation effects in vitro. Non‐excitatory CCM pulses delivered in the absolute refractory period of the paced cardiac cycle induced significantly stronger contractility increase in the coculture model as compared to cardiomyocytes alone, suggesting potential role of cardiac ganglia in CCM therapy mode of action.

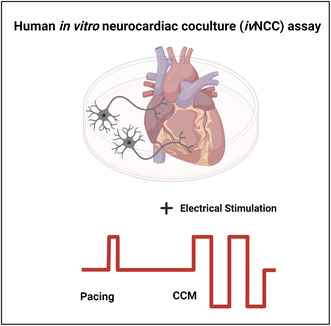

## 1 INTRODUCTION

Recent reports approximate about 6.2 million adults in the United States have heart failure and in 2018, heart failure was mentioned on 379,800 death certificates (Virani et al., 2020). The tridiagonal medical treatment strategy for systolic heart failure includes, but is not limited to, β‐blockers, angiotensin‐converting enzyme inhibitors, and mineralocorticoid receptor antagonists. Recent additions include sacubitril/valsartan and ivabradine for heart failure (Campbell et al., 2020; Narkar et al., 2022; Upadhya & Kitzman, 2020). Recently added to the treatment regimen is cardiac contractility modulation (CCM), an FDA approved intracardiac device therapy that targets patients with chronic heart failure, QRS <130 ms and a left ventricular ejection fraction (LVEF) 25%–45% who are currently receiving optimal medical therapy (OMT) yet remain symptomatic (Campbell et al., 2020; FDA.gov, 2019). While designing preclinical experiments and ultimately therapeutic strategy, one must rigorously investigate the inter‐organ communication and signaling. This allows to better mimic the physiological environment, cell‐to‐cell interactions and generates higher prediction value. Particularly in the case of the heart, there is a strong connection with the peripheral nervous system (Fedele & Brand, 2020; Kawano et al., 2003; Manolis et al., 2021; Rajendran et al., 2019; Winbo & Paterson, 2020; Zandstra et al., 2021).

The intrinsic cardiac nervous system (ICNS) is made up of afferent (sensory), interconnecting (local circuit), and cardio‐motor (efferent sympathetic and parasympathetic) neurons (Armour, 2008; Fedele & Brand, 2020). Using clearing‐imaging‐analysis pipeline in 3D heart models Rajendran et al., identified cholinergic and noradrenergic neurons in intrinsic cardiac ganglion and stellate ganglia that project to the sinoatrial node (Rajendran et al., 2019). The cardiac motor and sympathetic neurons are widely distributed throughout the cardiac chambers and spread through the intermediolateral cell column of the spinal cord and project their axons via C7–T6 rami to cardiac post‐ganglionic neuronal somata found in superior and middle cervical, mediastinal, and stellate ganglia (Armour, 2010; Coskun & Lombardo, 2016; Franzoso et al., 2016). The network of intracardiac ganglia and interconnecting neurons tightly modulate electrophysiology, conduction and thus are of key interest when studying arrhythmias and contractility modulation. For instance, in atrial fibrillation, simultaneous sympathetic and parasympathetic activations are the most common trigger. In contrast, in ventricular fibrillation in the setting of cardiac ischemia, sympathetic activation is proarrhythmic, whereas parasympathetic activation is antiarrhythmic (Shen & Zipes, 2014; Zandstra et al., 2021). In addition, specific neurocardiac pathologies affect both systems simultaneously, such as Huntington's disease, Lewy body diseases, Friedreich ataxia, congenital heart diseases, Danon disease, and Timothy syndrome (Coskun & Lombardo, 2016). Neurocardiac ablation and electrical stimulation is still an emerging modality and under investigation. However, a key rate limiting step is availability of physiologically relevant human in vitro models to evaluate cardiac medical device safety and effectiveness, which hinders the regulatory review process and results in the significant burden on animal models (Harris et al., 2013; Strauss & Blinova, 2017). We previously demonstrated acute CCM effects like transient contractility increase, enhanced lusitropy, and increased calcium sensitivity using 2D cardiomyocytes alone (Feaster et al., 2021), while this provides important insights into contractility mechanisms a limitation of the monoculture model is the lack of neuronal innervation and signaling. Thus, to further strengthen our model and extend our CCM findings, we worked toward assay development to standardize neuronal cell innervation to cardiomyocytes. One of the most groundbreaking advances in the last decade of biomedical research has been the development of human induced pluripotent stem cell (hiPSC) models for identification of disease mechanisms and drug discovery (Berry et al., 2018; Li et al., 2020; Shi et al., 2017). Use of patient‐specific iPSC derived cell types for 2D coculturing and engineered tissue assay development increases complexity and physiological relevance thus leading to improved clinical relevance (Chukwurah et al., 2019; Sharma et al., 2019; Yike Huang et al., 2020). This is critical particularly for human neuronal and cardiac cell types where obtaining primary human sample is extremely limited and invasive. High‐throughput data from preclinical studies employing human iPSC‐based cellular assays can reduce burden on expensive and laborious animal studies and aid in better design of strategies for medical treatment (Blinova et al., 2018; Bot et al., 2018; Carlson et al., 2013; Hortigon‐Vinagre et al., 2021; Moretti et al., 2013; Park et al., 2008). In parallel with drug treatment, device therapies can also be life‐sustaining. Cardiac contractility modulation is of benefit to patients with symptomatic heart failure on OMT and with normal or mildly prolonged QRS duration, thus providing support for the large complement of heart failure patients who do not have an indication for cardiac resynchronization therapy (CRT) (Abi‐Samra & Gutterman, 2016;Campbell et al., 2020; Lyon et al., 2013). CCM is a device‐based therapy that involves applying relatively high‐voltage (≈7.5 V), long‐duration (≈20 ms), biphasic nonexcitatory electric signals to the right ventricular septal wall during the absolute myocardial refractory period (Campbell et al., 2020; Lyon et al., 2013). It is believed that the implanted CCM device works by eliciting an acute increase in global contractility, as well as chronically producing a sustained improvement in quality of life, exercise tolerance, and heart failure symptoms (Anker et al., 2019; Francesco Giallauria et al., 2021). Despite the initial promising clinical results of CCM therapy, the complete mechanistic understanding of its mode of action is lacking. Here, we report a human in vitro neurocardiac coculture (*iv*NCC) model optimized to investigate, for the first time, CCM effects for two commercial cell types representing neurocardiac connection using cardiomyocytes (hiPSC‐CM) and motor neurons (hiPSC‐MNs) under comparable experimental conditions. Using these pacing and CCM parameters, we demonstrate significant changes in neurocardiac contractility during CCM stimulation.

## 2 MATERIALS AND METHODS

### 2.1 Cell culture and maintenance

Cryopreserved hiPSC‐CMs (iCell Cardiomyocytes^2^, Catalog # R1017, Fujifilm Cellular Dynamic, Inc.) and iCell® Motor Neurons (Catalog # R1051, Fujifilm Cellular Dynamic, Inc.) were thawed and plated according to the manufacturer's instruction. Density, substrate, media conditions, and days in vitro (DIV) were optimized as per assay endpoints and requirements. Briefly, 5000–10,000 viable commercial neurons were plated per well on 96‐ and 48‐well plate glass bottom MatTek (Catalog # P96G‐1.5‐5‐F) as monolayers precoated with 1:100 fibronectin (Catalog # 11051407001, Roche Diagnostics) for 7–14 days and media was changed every 48 h. Post formation of appropriate neuronal morphology (i.e., long and healthy neurites), commercial cardiomyocytes 50,000 viable cells were plated on top of the neurons. Cells were grown for 7–14 days at 37°C in iCell Cardiomyocyte Maintenance Medium (Cat# M1003), iCell® Neural Base Medium 1 (Catalog # M1010, Fujifilm Cellular Dynamic, Inc.) with supplements, coculture 50:50 media and media were changed every 48 h. All wells showed regularly contracting layers of cells 48 h after plating. For the flexible mattress, 1,500,000 cells were plated per well of a 6‐well plate and allowed to recover from cryopreservation at least 2 days at 37°C. Cells were then dissociated, each well was washed twice with 2X volume (i.e., 4 ml) with DPBS (Catalog # 14190–144, Gibco). 1 ml of TrypLE™ Express was added and cells were incubated for 15 min at 37°C to dissociate. M3 medium consisting of RPMI 1640 with glucose 11.1 mM (Invitrogen, cat# 11875); 2% B27 with insulin (Invitrogen, cat#17504–044); 1% Pen‐Strep (Invitrogen, cat#17504–044) was used to quench the TrypLE™ Express and cardiomyocytes along with neurons were collected in a 15‐ml conical tube and centrifuged at 200 g for 5 min at 25°C. Cells were then resuspended in a small volume of M3 (e.g., 2 ml), filtered with a 100‐μm filter, counted, and plated on Matrigel (Catalog # 356230, Corning) mattress substrate as previously described (Feaster et al., 2015, 2021). CMs to neuron ratios (10:1) were used for coculture assays. Cells were maintained according to manufacturer's recommendations at 37°C, with 5% CO_2_ in the incubator.

### 2.2 Pacing and CCM stimulation

To test the effect of electrical stimuli on neurocardiac cocultures, we used a commercial pulse generator and software AM‐ Systems (Model 4100, World Precision Instruments, Sarasota, FL) to stimulate cells with square wave electrical pacing pulses (i.e., monophasic) to modulate coculture beat frequency at 1 Hz (2 ms stimulus pulse duration). Both pacing and CCM electrical pulses were delivered through the same pair of electrodes, resulting in field stimulation of the hiPSC‐CMs as previously described in (Blinova et al., 2014; Feaster et al., 2021). Square wave electrical pacing pulses (i.e., monophasic) were generated and cells were paced at 1 Hz (2 ms stimulus pulse duration), 5 V (14 V/cm, 64 mA). CCM stimulation was delivered as two biphasic pulses of 5.14 ms pulse duration (20.56 ms total duration), 7 and 10 V (20 and 28 V/cm, 90 and 128 mA, phase amplitude), zero interphase interval. The delay between pacing pulses and CCM stimulation was 30 ms. CCM pulse parameters were comparable with the setting typically used in clinical practice (Campbell et al., 2020; Feaster et al., 2021). Varying CCM pulse amplitudes were tested to determine the optimal CCM experimental amplitude range of 7 and 10 V to enable robust CCM response in mono and cocultures.

### 2.3 Measurement of contractile properties

Video‐based Contractility Platform and Software (CellOPTIQ, Clyde Biosciences), based on pixel displacement, was used to measure hiPSC‐CM contractility (Sala et al., 2018). Briefly, mono and coculture cells were plated as described earlier on matrigel mattress substrates on 48‐well glass bottom plates. Cells were imaged directly in plates by an inverted fluorescence microscope (Zeiss) using 40× objective. A camera attached to the front port of the microscope was used for contraction acquisition. CCM experiments were performed in 300 μl of Tyrode's solution pre‐warmed to 37°C containing (in mmol/L): CaCl_2_ 0.5, NaCl 134, KCl 5.4, MgCl_2_ 1, glucose 10, and HEPES 10, pH adjusted to 7.4 with NaOH. Contractile properties including contraction amplitude, contraction slope, relaxation slope, time to peak 50%, time to peak 90%, time to baseline 50%, time to baseline 90%, contraction duration 10%, contraction duration 50%, and contraction duration 90% were evaluated (Feaster et al., 2021; Sala et al., 2018). Temperature, 37°C and 5% CO_2_ were maintained by an environmental control chamber.

### 2.4 Immunofluorescence

For cardiac troponin and peripherin immunofluorescence, cells were fixed in 4% paraformaldehyde in phosphate buffer solution (Thermo Fisher Scientific; Catalog # AAJ19943K2) for 15 min and permeabilized with 0.5% Triton X‐100. Blocking was done with 5% boiled normal goat serum in PBS/0.5% Triton X‐100. Primary and secondary antibodies were diluted in 2.5% (wt/vol) bovine serum albumin (BSA)/PBS/0.5% Triton X‐100. Specimens were incubated with primary antibodies Cardiac troponin (Catalog # MA5‐12960, Invitrogen), Peripherin (Catalog # AB1530, EMD Millipore) at 1:500 overnight, washed three times for 5–10 min, and incubated with fluorescently conjugated secondary antibodies for 2–4 h as described in detail elsewhere (Narkar et al., 2021). All washes were performed with PBS/0.5% Triton X‐100. DNA was counterstained with DAPI or Hoechst 33342 (Thermo Fisher Scientific). Vectashield (Vector Laboratories) was used for mounting. Troponin and peripherin images were acquired with the Olympus FV3000 Laser scanning microscope (20X air and 60X oil immersion objectives).

### 2.5 Drug treatments

All compounds were dissolved based on manufacturers' recommendations: nicotine (Sigma N1019), metoprolol (Sigma M5391). Each experiment typically measured pharmacological response for 3–6 wells taken from independent cell thawing and plating. Cells were incubated either with Maintenance Media, 50:50 coculture media, M3 media or with Tyrode's prewarmed to 37°C, solution containing (in mmol/L): CaCl_2_ 0.5, NaCl 134, KCl 5.4, MgCl_2_ 1, glucose 10, and HEPES 10, pH adjusted to 7.4 with NaOH with vehicle or drug and allowed to equilibrate for at least 5 min before experiments.

### 2.6 Statistical analysis

Prism 8 software (Prism 8, GraphPad Software, CA) was used for the statistical analysis. For evaluation of all cardiomyocyte beats in a given recording, or all beats in each group (i.e., before, CCM, and after) were combined and averaged. Since our pulse stimulator train for delivering pulses has time and train constraints, we performed averaging of beats. Our train and recording duration is 20 s, so for the paced cardiomyocytes we have 4–5 beats before CCM, 9 beats during CCM and after CCM stimulation we have about 6–7 beats. This method gives a more representative comparison for values before, during, and after CCM, unless you observe particular changes in contractility that are restricted to first beat during CCM. Differences among the groups are presented as mean ± standard error of the mean (SEM). Differences were assessed as percent change relative to before CCM using the standard two‐way ANOVA (Tukey's test), hypothetical values of zero. Results were considered statistically significant if the *p*‐value was less than 0.05.

## 3 RESULTS

### 3.1 Human in vitro neurocardiac coculture ( *iv*NCC) model characterization

**FIGURE 1:**
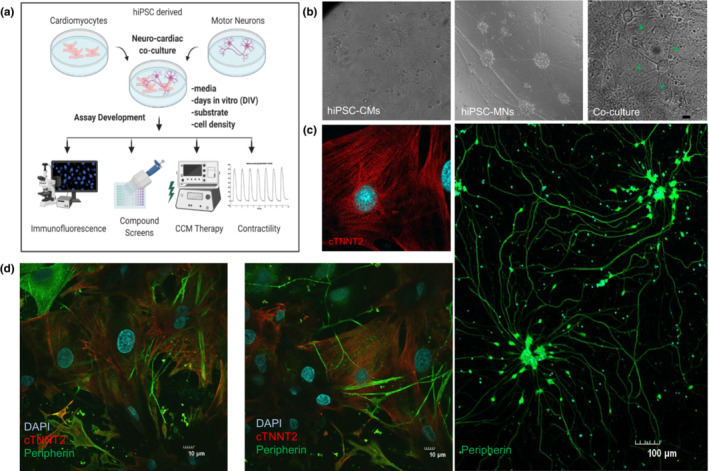
In vitro neurocardiac coculture (*iv*NCC) characterization. (a) Workflow schematic using hiPSC derived cardiomyocytes and neurons to optimize coculture assay parameters and endpoints. (b) Phase contrast images of cardiomyocytes alone, neurons and coculture (green arrowheads indicate long neurites). (c) Immunofluorescence staining of cardiomyocytes with cTNNT2 (left panel) and neurons with Peripherin (right panel) *n ≥ 6*. (d) Neurocardiac cocultures staining demonstrating colocalization of long neurites (green) with cardiomyocytes (red) in both panels.

We characterized optimal coculture parameters by testing various time points, cell number, DIV, substrate, media, and calcium concentrations. An overview of the workflow and assay development is illustrated in Figure 1a. The cocultures were viable on both matrigel and fibronectin substrates and optimal timepoints and assay windows ranged from 7 to 14 DIV. We tested neurocardiac cocultures for both, cardiac and neuronal markers using optical imaging. Phase contrast images of hiPSC‐CMs and hiPSC‐MNs alone as well as coculture show healthy morphology, monolayer formation, and progressive neurite outgrowth (Figure 1b). Immunofluorescence staining with cardiac troponin T (*TNNT2*) and peripheral nervous system marker peripherin (*PRPH*) showed significant expression (Figure 1c) indicative of successful differentiation. Cardiomyocytes demonstrate sarcomeric striations, flattened spread form monolayer syncytium and strong attachment and adhesion to substrates like matrigel and fibronectin. We observed elongated extension of processes from neurons reaching directly into hiPSC‐CMs, with neurites coursing over and through cardiomyocyte syncytia (Figure 1d). This confirmed that long neurite formations co‐localize with cardiomyocytes in coculture. Additionally, cardiomyocytes exhibited spontaneous beating in monoculture as well as in coculture starting Day 2 (Figure S1, Tables S3 and S4).

### 3.2 Coculture beating frequency can be modulated using electrical pacing

**FIGURE 2:**
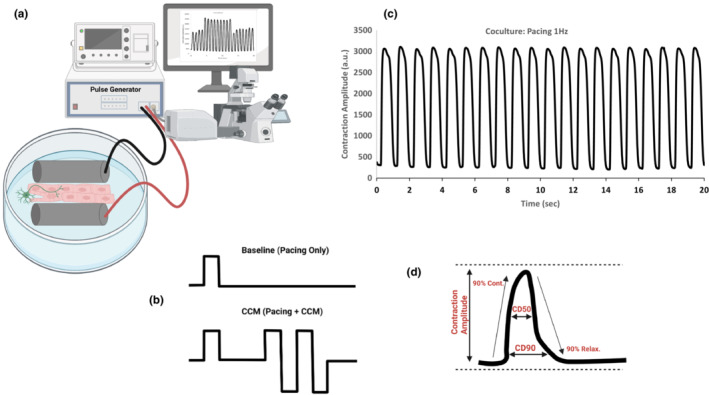
Neurocardiac coculture model for pacing and CCM therapy. (a) Setup of CCM coculture model using commercial pulse generator attached to platinum electrodes parallel to matrigel mattress along with contractility imaging. (b) Baseline pacing only waveform (top) and clinical CCM waveform (bottom) biphasic (7 V, 30 ms delay) for testing. (c) Representative contractility trace with 5 V pacing on coculture using 1 Hz frequency, recording 20 s with 40x objective, *n ≥ 6*. (d) Key contractility parameters recorded and analyzed shown with waveform illustration.

Using a pulse generator, electrical pacing was delivered to both, neurocardiac cocultures and cardiomyocyte monoculture. The setup shows commercial pulse generator attached to platinum electrodes placed parallel to matrigel mattress along with contractility imaging (Figure 2a). Figure 2b shows schematic waveforms used for either pacing (Pacing Only, single monophasic square pulse) or pacing accompanied by non‐excitatory CCM pulses (Pacing+CCM, single monophasic pacing pulse followed by two biphasic CCM pulse). Using 2D monolayer format on flexible substrate (i.e., Matrigel mattress) (Feaster et al., 2015) and submaximal extracellular calcium concentration [0.5 mM], we applied 1 Hz pacing pulses and observed neurocardiac beat rate at 60 beats per minute (bpm) (Figure 2c). These results demonstrate that coculture beat rate can be controlled by external electrical stimuli. Using these experimental conditions, acute CCM effect on contractility parameters including contraction amplitude (CtAmp), contraction time (UP90), relaxation time (Dn90), time to peak (TTP) (Figure 2d) were quantified in further experiments and analysis.

### 3.3 Acute CCM significantly increases coculture contractility amplitude

**FIGURE 3:**
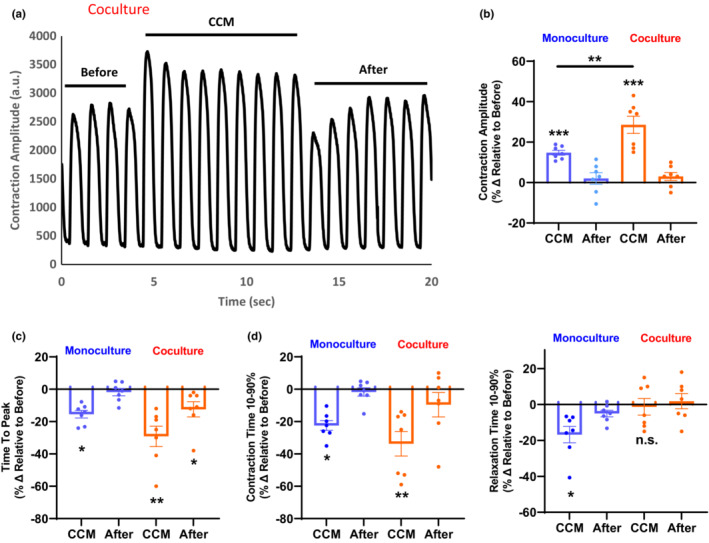
Acute effects of cardiac contractility modulation (CCM) on human induced pluripotent stem cell derived neurocardiac coculture contractile properties. (a) Representative contraction recording trace for coculture before (5 V), during CCM (7 V), and after (5 V). (b) Comparison analysis of quantification of contraction amplitude for monoculture v/s coculture during CCM. (c, d) Summary bar graphs of immediate contractility kinetics and effects including Time To Peak, Contraction Time (10%–90%) and Relaxation Time (10%–90%). Percent change shown is relative to the readings before CCM stimulation, data are mean ± SEM. *n* ≥ 6. *p < 0.05, **p < 0.01, ***p < 0.001, ****p < 0.0001, n.s. not significant.

To extend our previous observations (Feaster et al., 2021) and elucidate neuronal contribution to the CCM response we evaluated CCM stimulation on coculture and monoculture in parallel. Cardiomyocyte monoculture alone can be paced at 1 Hz using monophasic pulses (Figure S2a and Table S2). We also observed increased contraction amplitude in monoculture during CCM (Figure S2b), consistent with previously reported CCM‐induced changes (Harris et al., 2013). When exposed to pacing+CCM stimulation, the neurocardiac coculture exhibited significant changes in cardiac contractility that ceased when CCM signal was stopped. Typical contractility recording in *iv*NCC is shown in Figure 3a and includes three parts—before, during, and after CCM stimulation. There was a significant increase in contraction amplitude (29 ± 4%) relative to before application of CCM pulse (Figure 3a,b). Most importantly, the observed increase in amplitude during CCM in coculture was significantly greater than in monoculture alone (Figure 3b; Table S5). We also observed significantly faster contraction kinetics (Contraction time 10%–90%) and Time To Peak in both monoculture and coculture (Figure 3c). Relaxation Time (10%–90%) was significantly faster as compared to before CCM pulse application in monoculture but not in coculture (Figure 3d). These effects remained for the entire duration of CCM stimulation (Tables S1 and S2). These results demonstrate that acute CCM stimulation enhances hiPSC‐CMs contractile properties in presence of neurons to a greater extend as compared to cardiac monoculture.

### 3.4 Functional coupling in neurocardiac coculture increases cardiomyocyte beat rate

**FIGURE 4:**
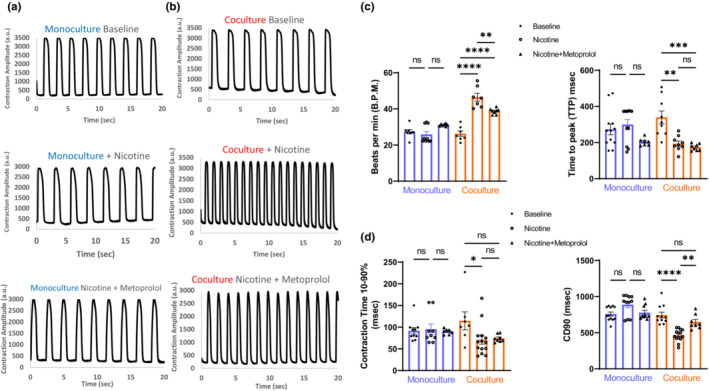
Functional coupling using nicotine and metoprolol. (a) Representative contractility traces at the baseline, after 10 min nicotine exposure and after addition of metoprolol in coculture. (b) Similar traces for monoculture. Summary bar graphs for contractility parameters including beat rate and kinetics like Time To Peak, UP90, and Contraction Duration CD90 for monoculture v/s coculture post nicotine alone and nicotine + metoprolol exposure. (c, d) Data are mean ± SEM. *n* ≥ 6. *p < 0.05, **p < 0.01, ***p < 0.001, ****p < 0.0001, n.s. not significant.

To confirm functional coupling of neural and cardiac cells in culture together, we used pharmacological challenge. We tested addition of presynaptic agonist nicotine 1 μM to mono and coculture. Nicotine can stimulate release of the β‐adrenergic agonist norepinephrine (NE) from neurons. This pathway can modulate contractility and beat rate through second messenger signaling cascade. We observed a significant increase in beat rate in coculture post‐acute treatment with nicotine (Figure 4a–c). There was a significant decrease in contractility parameters including Time to Peak, Contraction Time (UP90) and Contraction Duration (CD50) in coculture (Figure 4c,d). Addition of nicotine did not show significant changes in contractility parameters in monoculture alone (Figure 4b–d). Post nicotine treatment we also observed faster contraction kinetics including Contraction Duration (CD90), Interval, Relaxation Time (Dn90) that were significantly decreased in neurocardiac cocultures (Figure S3a–d), demonstrating that the presence of neurons was necessary to modulate cardiomyocytes contractility by nicotine. To further validate the coupling, we added Metoprolol 2 μM, a β‐adrenergic antagonist post nicotine treatment. We observed a partial but significant rescue in beat rate of coculture and CD90 (Figure 4c,d). Other contractility parameters like Interval and Relaxation Time were also significantly altered in coculture (Figure S3a,d). These data not only strengthen the colocalization and physical contact of cardiac and neural cells cultured together, but also support functional signaling that can modulate contractility potentially via the β‐adrenergic signaling pathway in the presence of functionally coupled neurons and suggest that ivNCC assay developed here might also be applicable to other experiments (beyond CCM), where cardiac ganglia/heart innervation is expected to play a role.

## 4 DISCUSSION

The novel human in vitro coculture model using commercially available iPSC‐CMs and neurons was established here and, for the first time, applied to the non‐clinical assessment of the cellular level effects of CCM stimulation. This more physiologically relevant neurocardiac model was used to demonstrate the potential contribution of direct neural stimulation during medical CCM therapy and to improve understanding of the CCM mechanism of action to support regulatory decision‐making for these novel devices. One major question arises regarding the mechanism by which non‐excitatory electric currents enhance myocardial contractility in vivo is whether increased contractility of the whole heart is a consequence of targeted regional effects or do these signals have more global effects involving regulation via the peripheral nervous system. An interesting observation from our work is that the contractility amplitude increase in coculture is significantly more pronounced than monoculture alone (Figure 3b) suggesting the involvement of neuronal innervation during electrical stimuli. We also noticed that as compared to earlier models like rabbit CMs, hiPSC‐CMs (Blinova et al., 2014; Feaster et al., 2021) the *iv*NCC displays sustained amplitude increase even after the first beat during CCM application. Further investigation is warranted into cardiac ion channels, calcium concentrations to understand this sustained effect. This effect returns near baseline, as was seen in earlier models once CCM stimulation is stopped, thus reinforcing that the effect is transient and appears to be directly related to electrical stimulation. This study evaluated only acute CCM treatment in coculture, however, chronic treatment with electrical stimulation is an area that is currently being investigated by our group.

Neurocardiac functional coupling can be validated by presence of synaptic varicosities, special pre‐ and post‐synaptic cellular membranes, receptors and gap junction expression on cardiomyocytes and neurotransmitter release via vesicles (Franzoso et al., 2016; Oh et al., 2016; Shen & Zipes, 2014; Winbo et al., 2020). Robust functional coupling of hiPSC‐derived neurons with target tissues in vitro is essential for modeling intercellular physiology in a dish and to further translational studies but it has proven difficult to achieve. Here, we successfully demonstrate colocalization by immunostaining and functional coupling by addition of nicotine that increases beat rate and alters neurocardiac contractility parameters (Figures 1 and 4). Our data also supports previous reports that have shown similar increase in coculture beating rate post nicotine exposure (Oh et al., 2016; Winbo et al., 2020). As this increase in beat rate is specific to neurocardiac cultures and not cardiomyocyte monoculture, it highlights the role of neuronal innervation in maintaining cardiac tone in presence of external stimuli like nicotine. Addition of metoprolol alters several contractility parameters including beat rate demonstrating a potential dependence on β‐adrenergic pathway for neurocardiac coculture. One of the biggest advantages of our *iv*NCC model is the use of commercial hiPSC derived cardiomyocytes and neurons that account for maximum reproducibility and can be easily optimized to investigate different human neuronal cell subtypes. This also helps overcome the low efficiency of reprogramming and heterogeneity issues. In the future, novel CCM devices are expected to be developed to address additional patient populations and neurocardiac device functionalities.

### 4.1 Study limitations

Listed below are the limitations of our study. We studied only acute effects of CCM on healthy commercial hiPSC derived cardiomyocytes and neurons and did not consider molecular and pathway changes within cardiomyocytes that occur in chronic heart failure. Modulating waveform, duration, delay and other pulse parameters can assist in determining combinations that can be beneficial in future studies for assessing in‐depth CCM effects. Although out of the scope of this study other factors like loss of sensitivity of tissue to prolonged stimulation, depletion of neurotransmitters and loss of natural cardiac autonomic tone need to be carefully investigated. With the simplified in vitro model one must factor in lack of chamber specific regulation by left and right stellate ganglia, limited inotropic responses and tissue architecture (Burnham et al., 2021; Yang et al., 2014). Different neuronal subtypes like sympathetic, parasympathetic may exert variable contractility amplitude depending on CCM pulse parameters and duration. Additionally, our model focuses on optimizing and assay development for *iv*NCC to test CCM effects on neurocardiac cocultures with limited investigation in mechanistic and molecular signaling pathways. Gene expression changes, protein abundance and mitochondrial effects, though beyond the scope of this study are warranted to better understand differences in neuronal subtypes and their downstream signaling mechanisms post CCM stimulation. Some other general limitations of in vitro iPSC models are discussed elsewhere in detail (Feaster et al., 2021; Liu et al., 2022).

### 4.2 Neuromodulation therapies (ANS, vagal stimulation)

The peripheral nervous system, particularly the two arms of the autonomic nervous system (Sympathetic and Parasympathetic) play a vital role in cardiac arrhythmogenesis. (Manolis et al., 2021; Shen & Zipes, 2014; Winbo & Paterson, 2020). Depending on pathology for instance atrial fibrillation (AF) and ventricular arrhythmias (VAs) neuronal innervation can be proarrhythmic or antiarrhythmic. This control can also be dysregulated in adrenergic or catecholamine‐sensitive arrhythmias, certain channelopathies, for example, Long QT syndrome (LQTS) and vagal arrhythmias like vagotonia, sinus bradycardia and Brugada syndrome (Coskun & Lombardo, 2016; Manolis et al., 2021; Zandstra et al., 2021). Thus, modulating autonomic nervous system (ANS) may lead to prevention and management of cardiac arrhythmias. Medical device and drug interventions targeting both the extrinsic and intrinsic cardiac nervous system must undergo rigorous validation and safety assessment. ANS‐modulating interventions like cardiac sympathetic denervation, renal denervation, vagal stimulation, ganglionated plexi ablation and optogenetics have been employed as investigative tools (Akdemir & Benditt, 2016; Kobayashi et al., 2013; Manolis et al., 2021). Our *iv*NCC model provides a platform for preclinical testing of novel CCM therapies and other neuromodulation tools. Local‐circuitry neurons exhibit redundant functioning that ensures stable control of cardiac motor neurons throughout each cardiac cycle. Our coculture models can be directly used to study multiple cardiac cycles during normal and ischemic events using diseased hiPSC models. Research on the therapeutic modulation of cardiac autonomic tone by electrical stimulation has yielded encouraging early clinical results (Akdemir & Benditt, 2016; De Ferrari et al., 2011; Kobayashi et al., 2013; Schwartz et al., 2008). Direct application of electrical stimuli as in the case of CCM therapy may help stabilize normal neuronal output function, leading to balance between cardiac cholinergic and adrenergic efferent neuronal outputs and thus contributing to suppression of arrhythmias. This coculture model can be extended to diseased backgrounds including HF and different neuropathies. As stated earlier, the sympathetic and parasympathetic autonomic systems affect cardiac function differently, but they can also have direct inhibitory effects on each other at the neurotransmitter level.

### 4.3 Electrical stimulation and neurotransmitter release

Mechanism of action by which electrical stimulation improves cardiac contractility appears multifactorial with acute changes in calcium handling, and chronic improvement in expression and phosphorylation of key calcium regulatory pathways (Abi‐Samra & Gutterman, 2016; Feaster et al., 2021; Lyon et al., 2013). The acute CCM effect is seen in isolated rabbit papillary muscle and also in trabecular muscle of certain patients with heart failure, where they observe a 50% increase over baseline contractility on exposure (Blinova et al., 2014; Daniel Burkhoff et al., 2001). A dog heart failure model also showed that 1 h CCM increased ejection fraction from 31% at baseline to ~41% (Morita et al., 2003). Cellular benefits of CCM involve upregulation of L‐type calcium channels and improvement of calcium uptake into the sarcoplasmic reticulum (SR), thereby enhancing extracellular calcium influx during the subsequent membrane depolarization along with calcium induced calcium release from the SR. Other contributors include but are not limited to upregulation of SERCA2A, phosphorylation of phospholamban, activation of L‐type calcium channels, restitution of the sodium/calcium exchanger, upregulation of metallomatrix proteins, gene expression changes, structural remodeling, and reduction in basement membrane fibrosis. (Butter et al., 2008; Imai et al., 2007; Lyon et al., 2013). More recently selective induction of ANS neurons is being actively investigated to understand cardiomyocyte proliferation, beat rate regulation and electrophysiological control. While our current *iv*NCC model is limited to only one type of peripheral neuron, other cocultures have tested compounds using sympathetic and/or parasympathetic neurons to gain better mechanistic understanding on potential neurotransmitters released (Oh et al., 2016; Takayama et al., 2020; Winbo et al., 2020). However, there are limited complex coculture models to test cardiac contractility modulation effects to assess device safety directly. The effects we observed in our *iv*NCC model may have an underlying amalgamation of structural and functional alterations like neurotransmitter regulation, tissue remodeling and gene expression changes in both neurons and cardiomyocytes during CCM pulse delivery. Although out of the scope of this study, a deeper dive into molecular insights before, during and after CCM stimulation is warranted. Towards this goal one can apply these coculture models to study acetylcholine and norepinephrine release during and after CCM stimulation. The tolerance of the cardiac plexus and vascular tissues to chronic CCM stimulation is yet to be studied in‐depth and may present as a tradeoff of effectiveness versus safety and long‐term stability.

## 5 CONCLUSIONS

This study opens exciting avenues in field of neurocardiology and provides an approach to generate pre‐clinical data that can supplement regulatory decision‐making process for evaluating future novel CCM devices and neuromodulatory therapies. Taking the above‐mentioned limitations into account, our work provides the first CCM characterization of two commercial hiPSC derived cell line models (i.e., hiPSC‐MNs and hiPSC‐CMs) exposed to the clinically relevant CCM stimulation conditions, using a novel *iv*NCC model. We observed elongated extension of neurite processes reaching directly into hiPSC‐CMs and coursing through cardiomyocyte syncytia. Successful colocalization, maturity marker expression and functional coupling was demonstrated using immunostaining, pharmacological assessment, and contractility assays. Neurocardiac coculture beat frequency can be modulated by pacing with electrical stimuli at 1 Hz. Lastly, we demonstrate significant increase in contraction amplitude and faster contraction kinetics in neurocardiac coculture during CCM stimulation as compared to monoculture alone. This characterization highlights the role of the cardiac ganglia in modulating contractility parameters during CCM electrical stimulation.

## DISCLAIMER

This article reflects the views of the authors and should not be construed to represent the US Food and Drug Administration's views or policies. The mention of commercial products, their sources, or their use in connection with material reported herein is not to be construed as either an actual or implied endorsement of such products by the Department of Health and Human Services.

## AUTHOR CONTRIBUTIONS

A.N., T.F. and K.B. conceived and designed research. A.N., T.F. performed experiments and analyzed data. A.N, T.F., M.C. and K.B interpreted results of experiments. A.N and K.B. drafted manuscript; M.C. and T.F. edited and revised manuscript. A.N., M.C., T.F., and K.B. approved final version of manuscript.

## FUNDING INFORMATION

The study was supported by the U.S. Food and Drug Administration, Office of Science and Engineering Laboratories and by the Center for Devices and Radiological Health.

## CONFLICT OF INTEREST

The authors declared no competing interest for this work.

## Supporting information


**Appendix S1:** Supporting Information
