# Supplementary material for: Human in vitro neurocardiac coculture (iv NCC) assay development for evaluating cardiac contractility modulation
Source: Physiol Rep. 2022 Nov 2;10(21):e15498. doi: 10.14814/phy2.15498 (PMC9630755; doi:10.14814/phy2.15498)
Supplement: Supplementary file 1 — Appendix S1: Supporting Information [file PHY2-10-e15498-s001.pdf]

Supplementary Figure Legends:

Supplemental Figure S1. Spontaneous contraction. **A**. Representative phase contrast image neurocardiac coculture with neurons in frame indicated by (white arrowhead). **B**. Representative spontaneous contractility trace for neurocardiac coculture on matrigel mattress n ≥ 6. Similar image and trace for monoculture alone on mattress (**C, D**).

Supplemental Figure S2. Monoculture pacing alone and CCM. A. Representative contractility trace of hiPSC-CMs paced at 5V, 1Hz frequency (monophasic) pulses and 2 ms pulse duration, recording 20 sec with 40x objective. **B**. Representative contraction trace recording for monoculture before (5V), during CCM (7V), and after (5V), n ≥ 6 collected with 20 sec recordings with 40x objective.

Supplemental Figure S3. Additional contractility changes post nicotine and metoprolol treatment. A. Quantification for contractility Interval changes post nicotine exposure and metoprolol in coculture and monoculture. **B**. Quantification for Contraction Duration (CD90) post nicotine exposure and metoprolol in coculture and monoculture. Similar summary bar graphs for contractility parameters including Contraction Amplitude and Relaxation Time Dn90 for monoculture and coculture (**C, D**). Data are mean ±SEM. n ≥ 6. *$p < 0.05$, $^{**}p < 0.01$, $^{***}p < 0.001$, $^{****}p < 0.0001$.

Supplementary Figures:

- **Figure S1.** Spontaneous contraction
- **Figure S2.** Monoculture Pacing alone and CCM
- **Figure S3.** Additional contractility changes post nicotine and metoprolol treatment

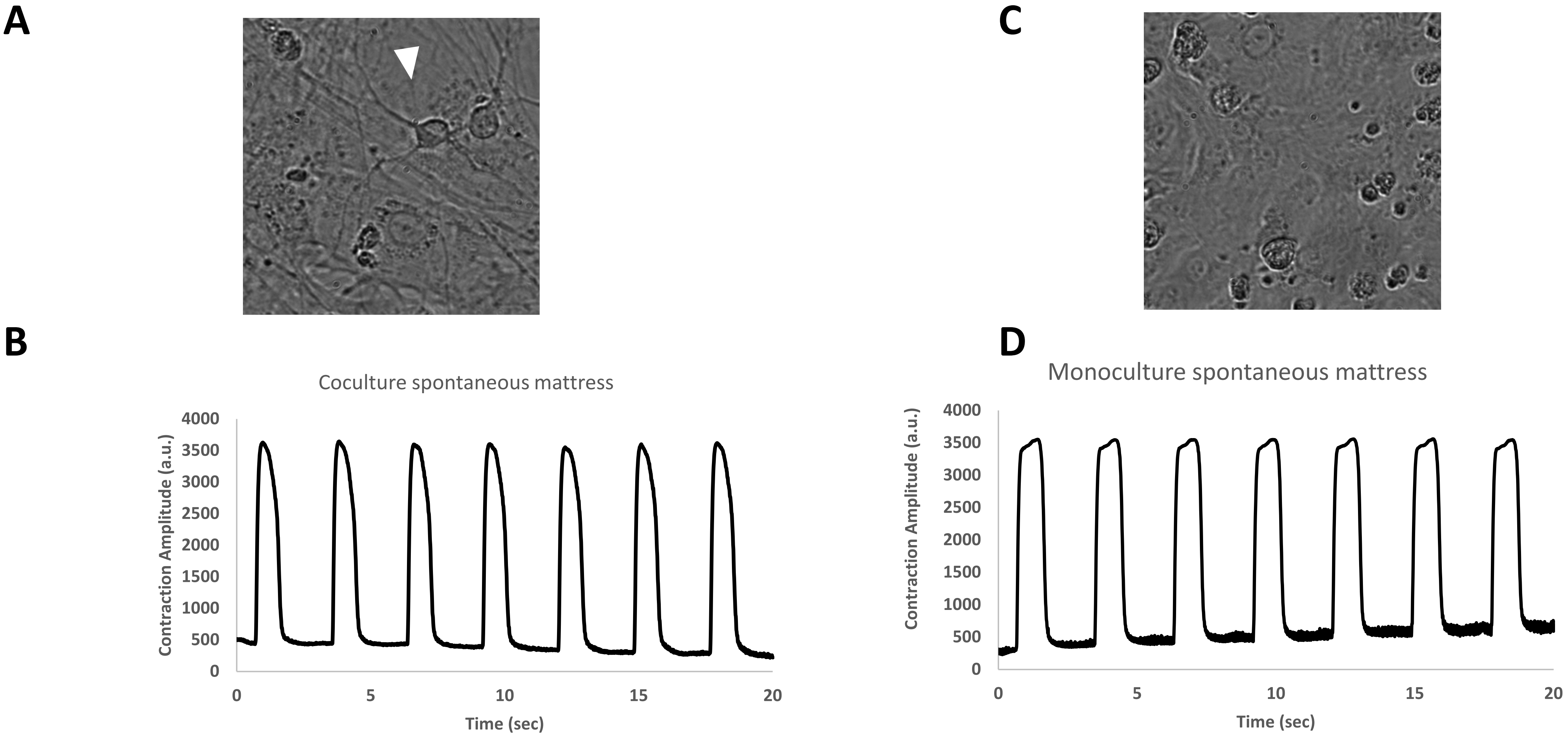
A
B
Coculture spontaneous mattress
4000
3500
3000
2500
2000
1500
1000
500
0
Contraction Amplitude (a.u.)
0
5
10
15
20
Time (sec)
C
D
Monoculture spontaneous mattress
4000
3500
3000
2500
2000
1500
1000
500
0
Contraction Amplitude (a.u.)
0
5
10
15
20
Time (sec)

Figure S1

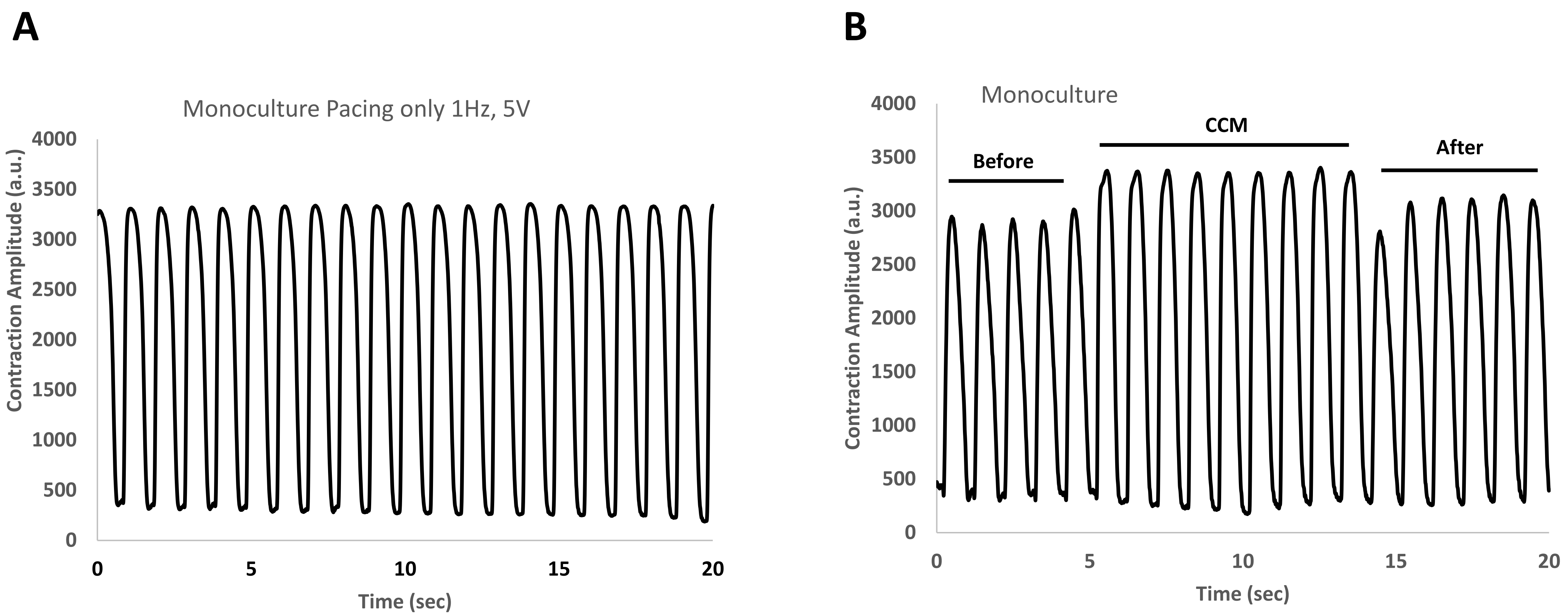
A
Monoculture Pacing only 1Hz, 5V
Contraction Amplitude (a.u.)
4000
3500
3000
2500
2000
1500
1000
500
0
0
5
10
15
20
Time (sec)
B
Monoculture
Before
CCM
After
Contraction Amplitude (a.u.)
4000
3500
3000
2500
2000
1500
1000
500
0
0
5
10
15
20
Time (sec)

Figure S2

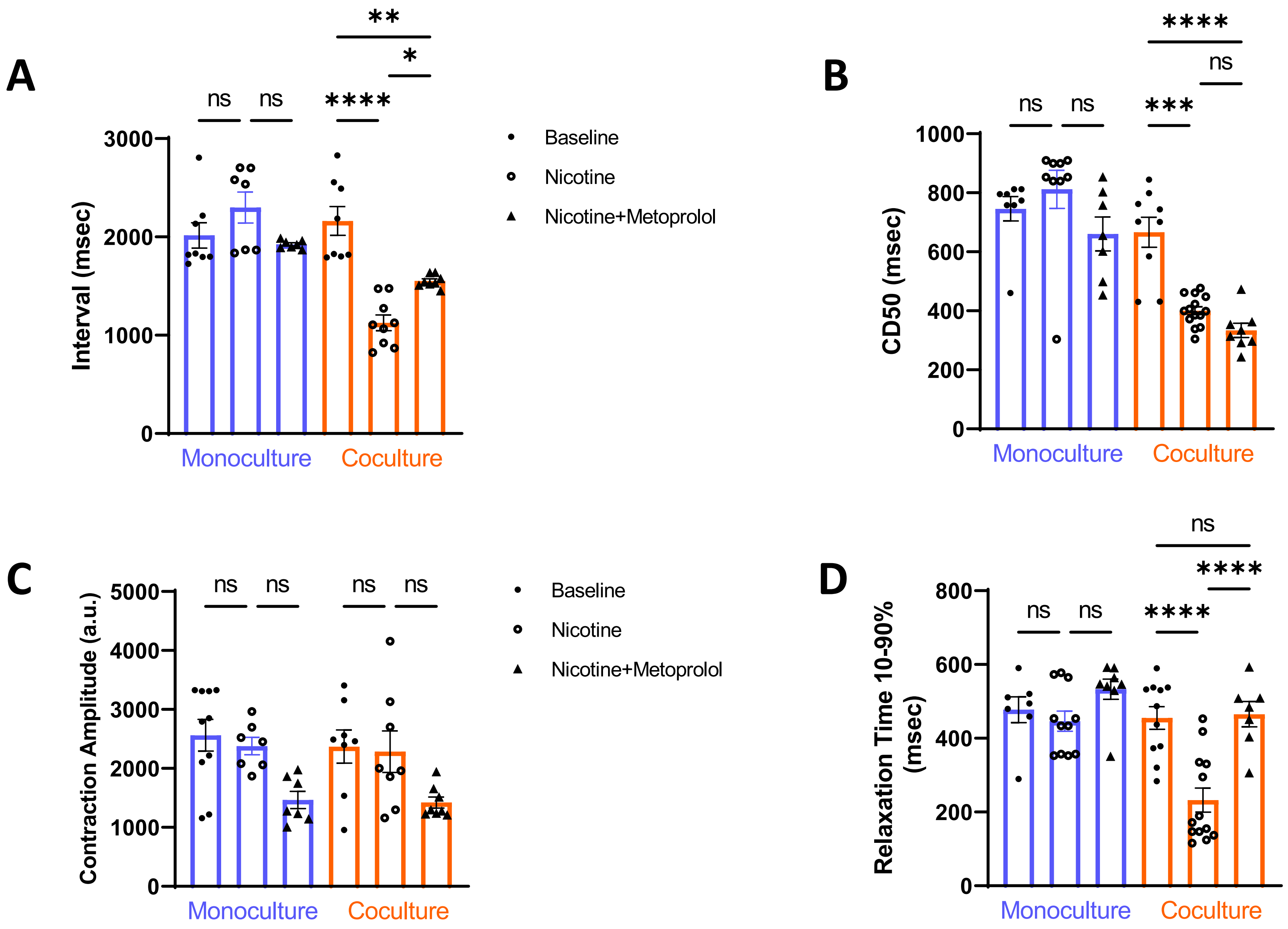
A
Interval (msec)
3000
2000
1000
0
ns
ns
****
*
**
Monoculture
Coculture
Baseline
Nicotine
Nicotine+Metoprolol
B
CD50 (msec)
1000
800
600
400
200
0
ns
ns
***
ns
****
Monoculture
Coculture
C
Contraction Amplitude (a.u.)
5000
4000
3000
2000
1000
0
ns
ns
ns
ns
Monoculture
Coculture
Baseline
Nicotine
Nicotine+Metoprolol
D
Relaxation Time 10-90%
(msec)
800
600
400
200
0
ns
ns
****
****
ns
Monoculture
Coculture

Figure S3

Supplementary Table Legends:

Table S1. Coculture CCM contractile properties. Percent change relative to before (5V), data are mean ± SEM for all beats in each group CCM (7V), and After (5V). n ≥ 7. *$P < 0.05$, **$P < 0.01$, ***$P < 0.001$, ****$P < 0.0001$.

Table S2. Monoculture CCM contractile properties. Percent change relative to before (5V), data are mean ± SEM for all beats in each group CCM (7V), and After (5V). n ≥ 7. *$P < 0.05$, **$P < 0.01$, ***$P < 0.001$, ****$P < 0.0001$.

Table S3. Baseline coculture contractility**.** Absolute values of baseline contraction, data are mean ± SEM; n ≥ 7, collected with 20 sec recordings with 40x objective.

Table S4. Baseline monoculture contractility**.** Absolute values of baseline contraction, data are mean ± SEM; n ≥ 7, collected with 20 sec recordings with 40x objective.

Table S5. Baseline contractility values for monoculture and coculture before CCM**.** Absolute raw contractility values for interval, amplitude, time to peak, contraction and relaxation time 10-90% at baseline before CCM; n ≥ 7, collected with 20 sec recordings with 40x objective.

Table S1. Coculture CCM Contractile Properties (Related to Figure 3)

**Parameter**	**CCM**	**After**
Amplitude	29 ± 4% ***	3 ± 2%
Time to Peak (TTP)	-15 ± 9% *	-7 ± 6%
TUp50	-26 ± 12% **	-4 ± 2%
TUp90	-27 ± 11% ***	-8 ± 5%
TDown50	-12 ± 1% *	-3 ± 1%
TDown90	-15 ± 5% *	-5 ± 2%
Contraction Duration CD10	-16 ± 6% *	-5 ± 3%
Contraction Duration CD50	-8 ± 1%	-3 ± 1%
Contraction Duration CD90	-4 ± 4%	0 ± 1%

Table S2. Monoculture CCM Contractile Properties (Related to Figure 3)

**Parameter**	**CCM**	**After**
Amplitude	14 ± 1% **	2 ± 2%
Time to Peak (TTP)	-6 ± 4% *	0 ± 2%
TUp50	-15 ± 6% *	-0.5 ± 3%
TUp90	-16 ± 5% **	-1 ± 2%
TDown50	-4 ± 1%	0 ± 1%
TDown90	-12 ± 2% *	0 ± 1%
Contraction Duration CD10	-0.65 ± 3%	-0.11 ± 1%
Contraction Duration CD50	-2 ± 1%	-0.9 ± 1%
Contraction Duration CD90	-4.7 ± 1%	3.3 ± 1%

Table S3. Baseline coculture contractility (Related to Figure 3, 4 and S1)

**Parameter**	**Values**
Amplitude (a.u.)	3111.8 ± 191.6
Time to Peak (TTP) (ms)	287.05 ± 11.9
Contraction Time Up90 (ms)	105.03 ± 7.6
Relaxation Time Dn90 (ms)	458.2 ± 23.6
TUp50 (ms)	93.08 ± 3.6
TUp90 (ms)	163.0 ± 8.6
TDown50 (ms)	802.9 ± 28.1
TDown90 (ms)	535.9 ± 28.6
Interval, Spontaneous (ms)	2448.7 ± 76.3
Beat Rate, Spontaneous (BPM)	24.8 ± 4.9
Contraction Duration CD10 (ms)	477.4 ± 29.5
Contraction Duration CD50 (ms)	709.8 ± 27.7
Contraction Duration CD90 (ms)	799.4 ± 31.5

Table S4. Baseline monoculture contractility (Related to Figure 3, 4 and S1)

**Parameter**	**Values**
Amplitude (a.u.)	2983.9 ± 94.6
Time to Peak (TTP) (ms)	388.0 ± 26.8
Contraction Time Up90 (ms)	176.3 ± 18.6
Relaxation Time Dn90 (ms)	373.6 ± 23.2
TUp50 (ms)	114.4 ± 5.9
TUp90 (ms)	235.6 ± 20.0
TDown50 (ms)	896.5 ± 7.5
TDown90 (ms)	661.5 ± 20.07
Interval, Spontaneous (ms)	2328.7 ± 61.6
Beat Rate, Spontaneous (BPM)	25.9 ± 3.9
Contraction Duration CD10 (ms)	602.3 ± 19.9
Contraction Duration CD50 (ms)	782.1 ± 11.01
Contraction Duration CD90 (ms)	799.5 ± 26.7

Table S5. Baseline contractility values for monoculture and coculture before CCM.

Before CCM (raw values)	Interval (msec)	Amplitude (a.u.)	Time to Peak (msec)	Contraction Time 10-90% (msec)	Relaxation Time 10-90% (msec)
Monoculture (Baseline)	1000	2749.5	310	141.2	377.1
	1000	4612.7	266.7	122.5	430.2
	1000	3532.6	290	141.3	410.4
	1000	832.5	220	98.4	205.1
	983.3	1420.3	210	117	209.7
	993.3	822.8	200	109.4	241.5
	1030	873.3	230	114.6	221.4
Coculture (Baseline)	990	2133	470	256.1	203.9
	986.7	852.1	503.3	143.8	192.8
	1003.3	1628.1	263.3	118.9	275.7
	1000	2583.1	253.3	112.7	367.1
	1003.3	2780	240	98.9	303.6
	1005	1859.4	350	162.9	251.6
	1010	1368.2	420	200.4	207.1
